# Exploring environmental risk in soils: Leveraging open data for non-sampling assessment?

**DOI:** 10.1016/j.heliyon.2024.e41247

**Published:** 2024-12-15

**Authors:** Silvia Aparisi-Navarro, Maria Moncho-Santonja, Beatriz Defez, Carla Candeias, Fernando Rocha, Guillermo Peris-Fajarnés

**Affiliations:** a Centro de Investigación en Tecnologias Gráficas. Universitat Politècnica de Valencia, Valencia, Spain; b GeoBioTec Research Unit, Geosciences Department, University of Aveiro, 3810-193, Aveiro, Portugal

**Keywords:** Heavy metal contamination assessment, Geostatistical raster maps, Heavy metal pollution indices, Soil sample data vs raster data, Comparison of contamination indices

## Abstract

Soil contamination by heavy metals (HM) is a critical area of research. Traditional methods involving sample collection and lab analysis are effective but costly and time-consuming. This study explores whether geostatistical analysis with GIS and open data can provide a faster, more precise, and cost-effective alternative for HM contamination assessment without extensive sampling.

Concentrations of nine HMs (Cu, Pb, Ni, Co, Mn, As, Cd, Sb, Cr) were analysed from 498 soil samples collected in two mining areas in Portugal: the Panasqueira and Aljustrel mines. Corresponding data were extracted from the Lucas TOPSOIL 1 km raster maps. Several contamination indices, Contamination Factor (Cf), Modified Contamination Degree (mCd), Geoaccumulation Index (Igeo), Nemerow Pollution Index (Pn), Potential Ecological Risk Index (PERI), and Pollution Load Index (PLI) were calculated for both datasets. A confusion matrix was used to evaluate the percentage of correct classifications, while a concordance analysis assessed the alignment of accurately classified points between the two data sources.

In the soil samples, very high contamination levels for As were observed in 42% of the samples, according to the Cf, with high levels for Sb found in approximately 30% of the samples. The mCd revealed that approximately 11% of soil samples exhibited very high levels of contamination, while the Pn indicated that 78.9% of the soil samples fell within the seriously polluted domain. Similar contamination trends were observed for the other indices. In contrast, the results for the LUCAS points showed significant discrepancies. No high contamination levels were found for any metal. The misclassification rates for mCd, Pn, PERI, and PLI were 84.25%, 97.55%, 95%, and 82%, respectively, when compared to the field data.

This study concludes that while open data raster maps offer rapid overviews, they fall short of providing the detailed precision required for reliable contamination assessments. The significant misclassification rates observed highlight the limitations of relying solely on these tools for critical environmental decisions. Consequently, traditional sampling and laboratory analysis remain indispensable for accurate risk assessments of HM contamination, ensuring a more reliable foundation for decision-making and environmental management.

## Graphical abstract

**Figure:**
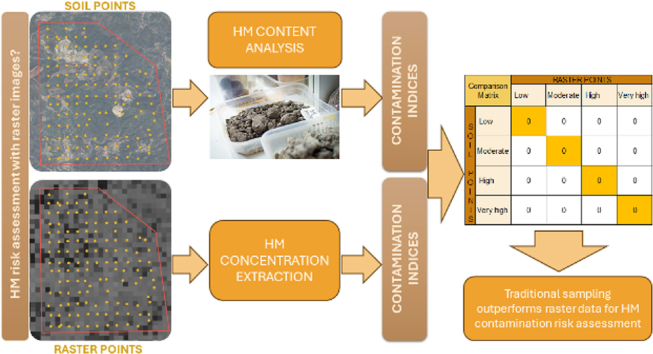

## 1 Introduction

Soil contamination by heavy metals (HMs) has garnered increasing attention in recent research due to their persistence and accumulation in ecosystems. HMs are resistant to degradation and can remain in soils for extended periods, with the potential to migrate into groundwater or crops, thereby impacting human health via the water supply and food chain [[1], [2], [3]].

HMs in soils can occur naturally or as a result of anthropogenic activities such as industrialisation, mining, and agricultural practices [4]. These sources have been widely studied in different contexts, including agricultural soils [[5], [6], [7]], urban environments [[8], [9], [10]], mining areas [11,12], lakes [13], and roadside soils [14,15].

HMs such as arsenic (As), cadmium (Cd), lead (Pb), zinc (Zn), and copper (Cu) are of particular concern due to their toxicity and persistence in the environment. Arsenic has been associated with various health problems, including skin, lung, and bladder cancers, cardiovascular diseases, and neurological disorders [16,17]. Similarly, cadmium exposure can lead to kidney diseases, bone damage, and pulmonary cancers [18]. Lead exposure affects the nervous and cardiovascular systems, even at low levels [19]. Although zinc and copper are essential trace elements, elevated levels can disrupt immune and cardiovascular functions [19,20].

HMs can enter ecosystems through various pathways, including industrial discharges, mining activities, and agricultural runoff. Natural geological processes also contribute to HM contamination in some regions [16]. Due to their toxicity and persistence, accurate risk assessments of HM contamination are essential for environmental management and public health protection.

Traditionally, the assessment of HM contamination has relied on direct sampling and laboratory analysis, which allow for accurate determination of HM concentrations and contamination indices [[21], [22], [23]]. When assessing larger areas, researchers often rely on data compiled from multiple publications [[24], [25], [26]]. However, the costs associated with these methods, which in Europe range from 5000 to 50000 euros [27], make large-scale studies both expensive and time-consuming [28].

To address these limitations, geostatistical methods based on geographic information systems (GIS) have been employed [[29], [30], [31]]. In particular, kriging models have been widely applied in environmental studies to estimate the spatial distribution of HM contamination without requiring extensive sampling [32,33]. These methods generate concentration maps over large areas, offering an efficient alternative to direct sampling[26,[34], [35], [36], [37]].

In Europe, one widely used dataset is the LUCAS Topsoil Survey, which provides maps of HM concentrations at a 1 km resolution based on a sampling density of 1 site per 200 km^2^ [38,39]. According to Hengl [40], this sampling level is suitable for creating continuous maps that provide reliable spatial representations of HM concentrations in the surface layer of European soils. These maps infer concentrations of As, Cd, Cr, Cu, Hg, Pb, Mn, Sb, Co, and Ni, with detection limits of 2.84, 0.07, 0.32, 0.26, 0.00005, 1.16, 2.12, 0.81, 0.15, and 0.27 mg kg^−1^, respectively, from over 20,000 specific sampling points [39].

**Fig. 1:**
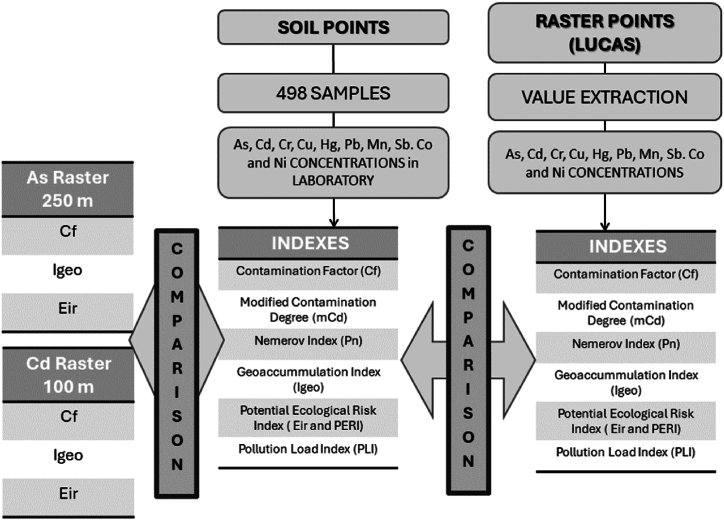
Working Scheme.

This study aims to evaluate whether open data, such as the LUCAS Topsoil Survey, can be used to conduct a rapid, cost-effective HM contamination risk assessment in specific areas without the need for extensive sampling. A total of 498 soil samples were collected from two mining regions, where HM contamination is expected to be significant [41,42]. Concentration levels of nine HMs (Cu, Pb, Ni, Co, Mn, As, Cd, Sb, Cr) were analysed, and contamination indices were calculated using both the soil samples and the LUCAS data. The results were compared to assess the accuracy of the LUCAS data in contamination risk assessment. Finally, to evaluate the influence of map resolution, maps with 100 m and 250 m resolutions were also compared (Fig. 1)

## 2 Material and methods

### 2.1 Study area

For this study, two locations were considered: the Panasqueira mine and the Aljustrel mine.

**Fig. 2:**
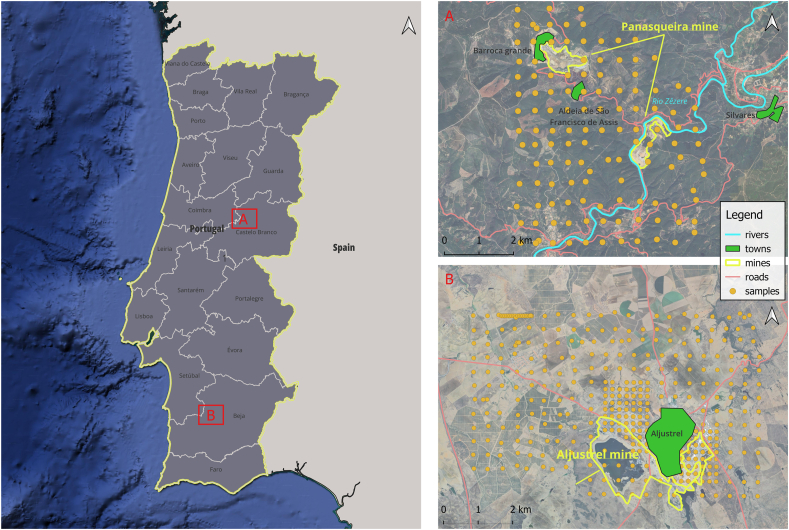
Location of the study areas: A) Panasqueira Mine B) Aljustrel Mine.

The Panasqueira mine is located in the central region of Portugal, in the district of Castelo Branco (606695, 4445577 UTM 29N). It is recognised as one of the largest SN-W deposits in Western Europe [43,44]. Elevations in the area range from 350 to 1080 m [45]. The climate is characterised by dry, hot summers and cold, rainy, and windy winters. Annual precipitation averages between 1200 and 1400 mm, with frequent snowfall. The average annual temperature is approximately 12 °C (Fig. 2A).

The Aljustrel mine is located in the Alentejo region in the district of Beja (574490, 4191926 UTM 29N). Aljustrel is one of the major mines in the Iberian Pyrite Belt [46]. The climate is Mediterranean, with warm temperatures and low precipitation compared to the national average. The average annual precipitation is 550 mm, with maximum temperatures reaching 40 °C in summer and 5 °C in winter (Fig. 2B).

### 2.2 Field sampling

A total of 489 samples were collected from the two sites (Fig. 2A and B). At Panasqueira, 126 samples were collected using a spaced grid pattern of approximately 400 m (Fig. 2A). The samples were collected at a depth of 0–15 cm, including sampling points both within and outside the mine area. Coordinates were determined using GPS and are georeferenced in UTM 29N. Further details on sample collection can be found in Ref. [47].

A total of 363 samples were collected in Aljustrel during two data collection campaigns (summer 2005 and summer 2006). Sample collection followed a grid pattern of 200 m in the contaminated area and 400 m in the rest of the area, resulting in a sampling density of 8 samples per km^2^. Coordinates were determined using GPS and are georeferenced in UTM 29N (Fig. 2B). Further details on sample collection can be found in Ref. [48].

### 2.3 Chemical analysis of soil samples

All soil samples were dried in an oven at 40 °C until reaching a constant weight, disaggregated, and passed through a 177 μm sieve (80 mesh), as this fraction likely best reflects metal contamination [49,50].

From each sample, 0–25 g were taken and heated in HNO_3_-HClO_4_-HF until fuming, then dried. Subsequently, the residue was dissolved in HCl and filtered. The solution underwent multi-element analysis at ACME Analytical Laboratories, an ISO 9002-accredited laboratory (Vancouver, Canada), using the inductively coupled plasma mass spectrometry (ICP-MS) method, with detection limits of Mn and Cr (1 mg kg^−1^), Cd, Cu, Ni, Co, Sb, and Pb (0.1 mg kg^−1^), and As (0.5 mg kg-1). Analytical precision and accuracy were assessed through the analysis of reference materials (C3 and G-2 standards) and duplicate samples in each analysis set. For a more detailed description of the sample collection and analysis procedure, refer to Refs. [47,48].

Hereafter, we refer to points whose HM concentrations were obtained through soil sampling and analysis as SOIL points.

### 2.4 Raster value extraction

To assess the feasibility of using open and high-resolution data for contamination risk assessment, concentration values of potentially toxic elements were extracted from each of the previous sampling points from the Europe HM maps [39] derived from the LUCAS TopSoil dataset [51]. Using the 'Raster Values Sample' tool in QGIS 3.28.4, the average concentration of each element (As, Co, Cd, Cr, Cu, Mn, Ni, Pb, and Sb) was obtained at each site. For details on the sampling and analysis methodology and subsequent map creation, refer to Refs. [38,39]. From this point forward, we refer to points whose HM concentrations were obtained through raster extraction as LUCAS points.

### 2.5 Environmental risk indices

In recent years, statistical methods have been extensively used to analyse the concentration, accumulation, and distribution of potentially toxic elements in soils [[52], [53], [54]]. Indices have been relied upon to interpret the degree of soil contamination, as they synthesise information [55] and effectively assess it [56]. In the case of mining areas, the use of indices is widely adopted as they monitor soil quality and support efforts towards long-term sustainability[[57], [58], [59]].

The following indices were calculated for both SOIL points and LUCAS points: Contamination Degree, Contamination Factor, Nemerov index, Geoaccumulation index, Potential Ecological Risk index, and Pollution Load Index.

#### 2.5.1 Contamination factor

One way to understand the level of soil contamination is through the contamination factor [60,61]. This index, as proposed by Hakanson [62], calculates a contamination factor (C_f_) for each studied contaminant. It is obtained by dividing the average concentration of each element in the soil (C_i_) by the average reference (or background) value of that element in the soil (C_b_). Equation (1)

(1) $$C_{f}=\frac{C_{i}}{C_{b}}$$

In this study, the reference values (mg kg^−1^) for soil were taken from the values provided by Reimann and de Caritat [63]: 5, 0.3, 10, 80, 25, 20, 17, 0.5, and 530 mg kg^−1^ for As, Cd, Co, Cr, Cu, Ni, Pb, Sb, and Mn respectively. The interpretation of the Cf can be found in Table 1.

**Table 1:** Interpretation of pollution indices.

Index	Value	Interpretation
*Contamination Factor (C*_*f*_*)*	*C*_*f*_*< 1*	*Low*
	*1 ≤ C*_*f*_*< 3*	*Moderate*
	*3 ≤ C*_*f*_*< 6*	*High*
	*6 ≤ C*_*f*_	*Very high*
*Modified Contamination Degree (mCd)*	*mCd < 1.5*	*Nil to very low degree of contamination*
	*1.5 ≤ mCd < 2*	*Low degree of contamination*
	*2 ≤ mCd < 4*	*Moderate degree of contamination*
	*4 ≤ mCd < 8*	*High degree of contamination*
	*8 ≤ mCd < 16*	*Very high degree of contamination*
	*16 ≤ mCd < 32*	*Extremely high degree of contamination*
	*mCd ≥ 32*	*Ultra-high degree of contamination*
*Nemerow Index (Pn)*	*Pn ≤ 0.7*	*Safety domain*
	*0.7 < Pn ≤ 1*	*Precaution domain*
	*1 < Pn ≤ 2*	*Slightly polluted domain*
	*2 < Pn ≤ 3*	*Moderately polluted domain*
	*Pn > 3*	*Seriously polluted domain*
*Geoaccumulation Index (Igeo)*	*Igeo ≤ 0*	*Unpolluted*
	*0 ≤ Igeo < 1*	*Unpolluted to moderately polluted*
	*1 ≤ Igeo < 2*	*Moderately polluted*
	*2 ≤ Igeo < 3*	*Moderately to heavily polluted*
	*3 ≤ Igeo < 4*	*Heavily polluted*
	*4 ≤ Igeo < 5*	*Heavily to extremely polluted*
	*Igeo ≥ 5*	*Extremely polluted.*
*Pollution Load Index*	*PLI > 1*	*Polluted*
	*PLI = 1*	*Baseline levels of pollution*
	*PLI < 1*	*Not polluted*

#### 2.5.2 Modified contamination degree

In addition to the contamination factor, Hakanson [62] proposed a general contamination indicator calculated from seven HMs (As, Cd, Cu, Cr, Hg, Pb, Zn) and the organic contaminant polychlorinated biphenyls (PCB) from five soil samples. This study proposed that the numerical sum of the eight contamination factors specific to each plot express the overall degree of soil contamination [64,65]. The classification proposed, and the calculation formula are based on and conditioned to these 8 study parameters defined by Hakanson and the five samples to calculate a correct and interpretable contamination degree.

Given the limitations of this index [66,67], the modified and generalised contamination index was introduced by Abrahim [68], and its calculation is performed according to equation (2).

(2) $${mC}_{f}=\frac{\left(\sum_{i=1}^{i=n}C_{f}\right)}{n}$$

Where n is the number of contaminants analysed, and C_f_ is the contamination factor. This generalised formula allows the incorporation of as many contaminants as required by the study. These contaminants can include both HMs and organic pollutants [69,70]. The interpretation of the modified contamination factor can be found in Table 1.

#### 2.5.3 Nemerow Index

The Contamination Index (PI) and the Nemerow Index (Pn) assess the level of contamination for each of the potentially toxic elements studied, as well as their integrated contamination effect, respectively [71,72]. It fully represents the amount of contamination at a specific location [73,74]. The Contamination Index PI is calculated using equation (3).

(3) $$PI=\frac{C_{i}}{S_{i}}$$

Where C_i_ is the average concentration of each element in the soil, and S_i_ is the background evaluation standard value of the element in the soil [[75], [76], [77]]. The S_i_ values have been obtained from Ref. [63]. The Nemerow index is calculated according to the following equation (4).

(4) $$P_{n}=\sqrt{\frac{{\left(\frac{1}{n}\sum_{i}^{n}{PI}_{i}\right)}^{2}+{\left(\mathrm{Max}\;PI\right)}^{2}}{2}}$$

Pn is the Nemerow contamination index, PI_i_ is the contamination index for an element at a specific site, and Max PI is the maximum contamination index for the metal [[78], [79], [80]]. The interpretation of the Nemerow Index can be found in Table 1.

#### 2.5.4 Geoaccumulation index

The Geoaccumulation Index (Igeo) by Muller [81] was initially employed in sediment evaluation in riverbeds; however, it can also be applied to assess soil contamination [82,83]. In fact, it has been widely used in assessing HM contamination in agricultural and urban soils [[84], [85], [86], [87], [88], [89], [90]], and it is calculated using equation (5):

(5) $$Igeo=\log_{2}\frac{Cn}{1,5\;Bn}$$

Where Cn is the measured concentration of the HM, Bn is the geochemical background concentration value, and 1,5 is the correction factor for the background matrix due to lithogenic effects [91,92]. The interpretation of the Igeo classification can be found in Table 1.

#### 2.5.5 Potential ecological risk index (PERI)

Hakanson [62] defined the potential ecological risk using equation (6):

(6) $$\begin{array}{c} Eir=T_{r}^{i}\times C_{f}^{i}\\ C_{f}^{i}=C_{iD}/C_{iB}\\ \;RI=\sum_{i=1}^{n}E_{r}^{i} \end{array}\;\;\;$$

Where n is the number of potentially toxic elements studied, C_f_^i^ is the contamination factor for each element, Eir is the potential ecological risk index of a single element, and T_r_^i^ is the toxicity coefficient of trace metal i. The toxicity coefficients for As, Cd, Cr, Cu, and Pb are 10, 30, 2, 5, and 5, respectively [62], 5 for Co [93], 5 and 1 for Ni and Mn [94] and 7 for Sb [95].

The classification of Eir and the risk index (RI) defined by Hakanson [62] is based on the maximum toxicity of 8 types of pollutants and the sum of the total toxicity of the eight elements studied [96]. To correctly interpret the indices when studying a different number of pollutants, it is necessary to adjust the threshold values between classes [[97], [98], [99]].

The first level of the Eir classification is defined assuming that the lowest contamination factor that can be obtained is 1 and that the Tir for the most toxic element studied is 30 (Cd). This means that even if there is no contamination (CF = 1), the Eir can reach a value of 30 [100,101]. The value range of each classification level is twice that of the previous range [96]. Therefore, the ecological risk index for a single element can be evaluated according to the following classification: Eir <30 low risk; 30 < Eir <60 moderate risk; 60 < Eir <120 considerable risk; 120 < Eir <240 high risk; Eir >240 significantly high risk.

To adjust the RI, the first classification value of the toxicity coefficient is determined as RI = 150 (limit value of the first classification grade of Hakanson)/133 (sum of the toxic coefficient of the eight contaminants studied by Hakanson) = 1.13. The sum of the toxicity coefficients of the nine elements in this study is 70. Therefore, to calculate the first classification level RI = 70 ∗ 1.13 ≈ 80. The value range of each classification level is twice that of the previous range [102]. In Table 2, the new classification limits calculated can be observed.

**Table 2:** Interpretation of the potential ecological risk index (PERI).

Classification	Value	New calculated values for 9 elements
*low risk*	*RI < 150*	*RI < 80*
*moderate risk*	*150 RI < 300*	*80 RI < 160*
*considerable risk*	*300 RI < 600*	*160 RI < 320*
*high risk*	*>= RI 600*	*>= RI 320*

#### 2.5.6 Pollution Load Index

The Pollution Load Index (PLI) was developed by Tomlinson [103] and consists of the geometric mean of the concentration C_f_ values of the n studied metals [104]. According to equation (7).

(7) $$PLI={\left({C_{f}}_{1}\times {C_{f}}_{2}\times .\;.\;.\times {C_{f}}_{n}\right)}^{1/n}$$

This index allows for a straightforward assessment of HM contamination levels. PLI values of 1 indicate the presence of HMs close to background levels, while values above 1 indicate contamination [105,106].

## 3 Results

The most significant results for the different contamination indices are presented. Refer to the supplementary material for a complete description of the results, including detailed data.

### 3.1 Contamination factor (C_f_)

In the SOIL samples, a very high contamination (C_f_ ≥ 6) (see Table 1) was observed for As in 42 % of samples, followed by Sb (27%), Pb (27%), Cu (13.5%), Mn (3.5%), and Cd (2.7%). High contamination (3 ≤ C_f_ < 6) was found mainly for Sb (37.6%) and As (31.7%). In contrast, none of the metals presented either very high or high contamination for the LUCAS points. Most were classified as low contamination (C_f_ < 1).

### 3.2 Modified contamination degree (Mcd)

For SOIL points, 15.75% of the samples showed nil or very low contamination, 25.15% low contamination, and 36.2% moderate contamination, with 2.25% classified as having ultra-high contamination. By comparison, all LUCAS points presented either nil or very low contamination. See Table 1 for the interpretation of the index categories.

### 3.3 Nemerov index (Pn)

The Nemerov Index calculations for SOIL points revealed that 78.9% of the samples were classified in the seriously polluted domain, 18.6% in the moderately polluted domain, and 2.5 % in the slightly polluted domain. In contrast, LUCAS points were mainly classified as slightly polluted (77.3%), with the remaining 22.7% categorised under the precaution domain.

### 3.4 Geoaccumulation index (Igeo)

**Fig. 3:**
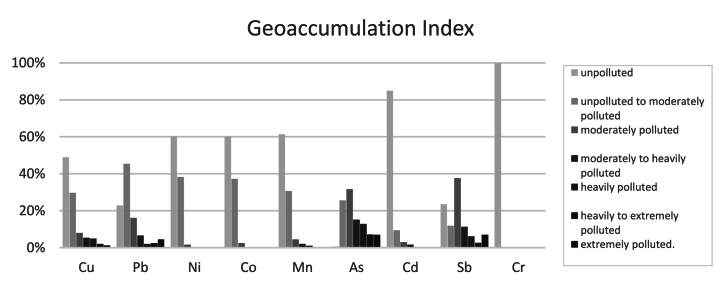
Results of the geoaccumulation index for soil points.

For the SOIL points, most sites were unpolluted for Cr (99.80%), Cd (84.87%), Mn (61.35%), Ni (60.12%), and Co (60.12%), with lower levels for Cu, Sb, Pb, and As. (Fig. 3).

For the LUCAS points, all metals were classified as non-contaminated, except for Nickel, where 34.2% were unpolluted (Igeo <0) and the rest were classified as unpolluted to moderately polluted (0 ≤ Igeo <1).

### 3.5 Potential ecological risk index (Eir and RI)

The SOIL data regarding (RI) indicated that 5% of the samples showed low risk, 53% moderate risk, 22% considerable risk, and 20% high risk. Meanwhile, the LUCAS data indicated that 99% of the samples showed low ecological risk.

#### 3.5.1 Pollution Load Index (PLI)

The LUCAS points were classified as non-contaminated for 100% of the samples. In contrast, SOIL points indicated contamination in 82% of the samples.

### 3.6 Summary of results

To assess the accuracy of using raster data for environmental risk assessment compared to soil sample data, confusion matrices were used. These matrices were constructed by calculating contamination indices for both datasets (soil samples and LUCAS data). The confusion matrices compare the correct classifications, where the diagonals represent the well-classified points. Classification accuracy was calculated by determining the percentage of points correctly classified based on soil samples, which were taken as the reference.

A concordance analysis was performed to evaluate the alignment of correctly classified points between both data sources, revealing discrepancies between LUCAS data and soil samples. This analysis highlights whether open data can serve as a reliable alternative to traditional soil sampling in environmental risk assessments.

The accuracy of using raster data for environmental risk assessment is summarised in Table 3, Table 4. For individual indices, the contamination factor (C_f_) showed the highest accuracy for Cr (89%) and Ni (74%), while As had no well-classified samples. For the Geoaccumulation Index (Igeo), Cr had 100% well-classified samples, while As had only 1%.

**Table 3:** % Success rate in classification for individual indices.

% success rate in classification	C_f_	Igeo	Eir
Cu	19	48	87
Pb	7	23	84
Ni	74	58	100
Co	34	59	100
Mn	53	61	100
As	0	1	21
Cd	58	85	58
Sb	18	24	55
Cr	89	100	52

Classification accuracy was lower for complex indices such as the modified contamination degree (mCd) and Nemerov index (Pn), with 15.75% and 2.45%, respectively.

**Table 4:** Success rate in classification for complex indices.

Index	% Success rate in classification
Mcd	15,75 %
Pn	2,45 %
RI	5 %
PLI	18 %

If we compare the Potential Ecological Risk Index (RI) between soil and LUCAS data, only 5% of the samples were well-classified. For the Pollution Load Index, 18% of the samples were correctly classified.

### 3.7 Do results improve with increased resolution?

To assess whether increasing the resolution improves the results of index calculations, raster values from the arsenic map (250 m pixel size) [107] and the cadmium map (100 m pixel size) [108] were extracted for the same soil points. Individual indices such as the Contamination Factor (C_f_), Geoaccumulation Index (Igeo), and Potential Ecological Risk Index for a Single Element (Eir) were calculated.

For arsenic, 26% of the samples were classified as low contamination, and the remaining 74% as moderate contamination according to the Contamination Factor (C_f_). For cadmium, 87% of the samples were classified as low contamination, with the rest categorised as moderate contamination.

The Geoaccumulation Index (Igeo) classified all samples as contamination-free for both arsenic and cadmium.

For the Potential Ecological Risk Index (Eir), 100% of the samples for arsenic were classified as low risk, while for cadmium, 87% were classified as low risk and the rest as moderate risk.

**Fig. 4:**
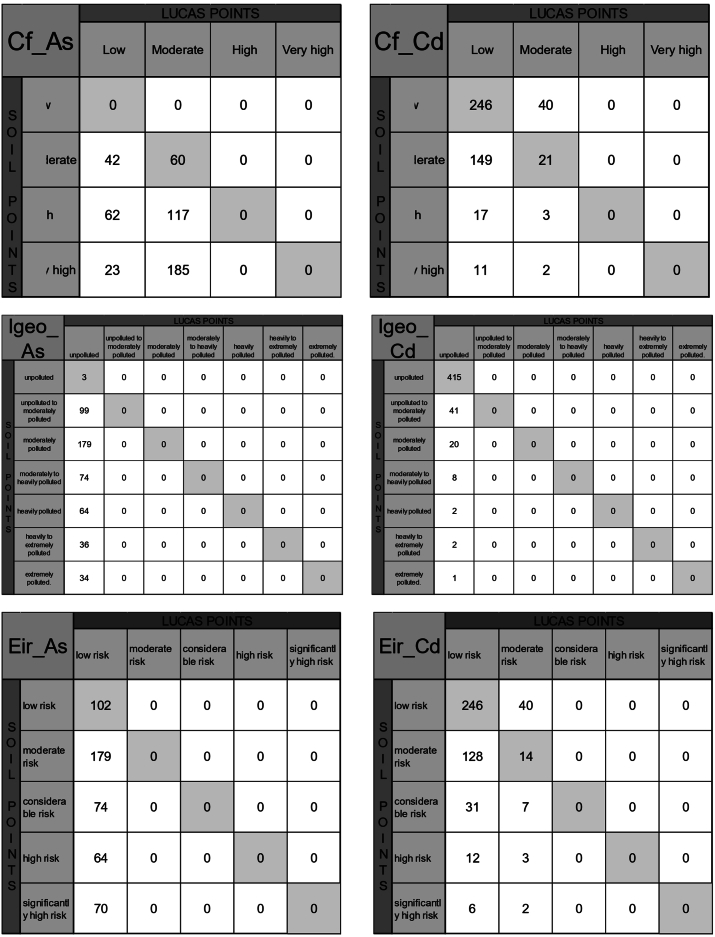
Contamination factor, Geoaccummulation Index and potential risk index of a single element comparison matrices for arsenic (250 m) and cadmium (100 m).

When comparing the classifications obtained from soil points with those from the LUCAS points (250 m for arsenic and 100 m for cadmium), 12% of the samples for arsenic and 55% for cadmium were well-classified for the Contamination Factor (C_f_). For the Geoaccumulation Index (Igeo), only 1% of the samples for arsenic were well-classified, while 85% of the samples for cadmium were correctly classified. Finally, for the Potential Ecological Risk Index (Eir), 21% of the samples for arsenic and 53% of the samples for cadmium were well-classified (Refer to Fig. 4 for matrix comparisons).

## 4 Discussion

**Fig. 5:**
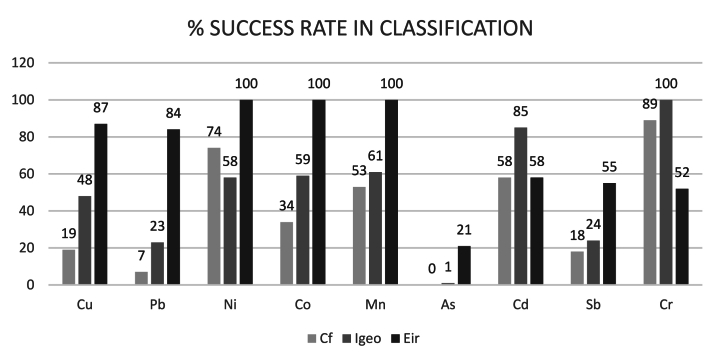
Success rate in classification for individual indices.

This study aimed to determine whether open raster data can be reliably used for environmental risk assessments. Initial analysis suggests that for some metals, open data could be useful when using specific individual indices, such as the Ecological Risk Index (Eir) for Ni, Co, and Mn or the Geoaccumulation Index (Igeo) for Cr or Cd (see Fig. 5). However, a deeper analysis highlights several limitations that challenge the reliability of these interpolated maps for detailed risk assessment.

When interpreting the results, it is crucial to consider the accuracy of HM raster maps. These maps are generated from predictions based on limited sampling points, which inherently introduces limitations and potential errors [109]. Moreover, the reliability of HM raster maps is affected by the detection limits of the analytical methods used during sampling [107].

In the case of arsenic, over 95% of the samples used for generating the LUCAS map contained less than 20 mg kg^−1^, with the majority falling below the detection threshold [39]. This limitation significantly affects the accuracy of raster maps when assessing soil contamination. As reflected in our study, arsenic had some of the lowest classification success rates, with just 0 % for the C_f_ index, 1% for Igeo, and 21% for Eir, underscoring the difficulties in identifying arsenic contamination using these maps.

The success rates of LUCAS data compared to soil-specific data may appear high for non-contaminated areas, as the indices tend to classify uncontaminated parcels correctly in both soil samples and LUCAS data. For example, the Eir index shows a success rate of 100% for Ni, Co, and Mn, reflecting its effectiveness in identifying non-contaminated soils for these metals. However, these indices lack the sensitivity needed to detect contaminated parcels adequately. While non-contaminated parcels are correctly classified, contaminated parcels appear as non-contaminated in the LUCAS data. This issue is evident for elements like As, where the success rate falls to only 21%, underscoring the inadequacy of these indices in identifying contamination hotspots.

An important factor to consider is the spatial resolution of the maps. Typically, higher resolution leads to more precise estimations [110], suggesting that better spatial resolution could improve risk assessment classification. However, in our case study, it is not the resolution of the LUCAS maps (whether 1 km, 250 m, or 100 m) that is the primary influence, but rather the sampling size, which determines the ability to capture small-scale variability in the predictive maps [40,111]. Moreover, the number of sampling points and their spatial distribution also significantly impact the quality of the generated map [112]. If these points were not specifically selected to assess contamination in the study area, it is unlikely that the LUCAS maps will accurately represent contamination levels in this region.

Data interpolation typically maintains the overall trends of the original dataset [113], allowing low-resolution, wide-coverage maps to provide a general perspective on a given issue without offering granular detail. However, this raises the question of how much sampling is necessary to generate an accurate map for a specific study area. Increased sampling density improves the performance of interpolation models. Considering that the LUCAS maps include just one sampling point per 200 km^2^, while our study areas—Panasqueira with 36 km^2^ and the second site with 48 km^2^—are significantly smaller, it becomes evident that, in the worst-case scenario, no sampling points would fall within the study boundaries. For contamination studies in this specific region, neither the data resolution nor the sampling density is sufficient to conduct a reliable risk assessment.

When reviewing previous studies on the Panasqueira mining area [114], analysed contamination using the Contamination Factor and the Modified Contamination Degree (mCd). Their findings indicated that the highest contamination levels were for arsenic (As), with moderately elevated levels for copper (Cu) and manganese (Mn), a pattern that aligns with the results observed in our study. In terms of the modified contamination degree, their study reported that 34.6% of the samples had an mCd <2, while in our case, we observed a slightly higher percentage, with 40.9% of the samples showing similar contamination degrees.

Several studies have investigated contamination levels around the Panasqueira mine [115]. analysed HMs in plants near the mine, finding elevated concentrations of arsenic (As) and copper (Cu) exceeding the limits recommended by the World Health Organisation.

Similarly [116], assessed the potential ecological risk in rhizosphere soils, reporting that 12.9% of the sampling sites showed very high ecological risk, 27.7% considerable risk, 35.2% moderate risk, and 24.1% low risk. Furthermore [117], concluded that soils near Panasqueira were heavily contaminated with As, Cd, Cu, and Zn. These findings align with our observations of metal contamination in the area, further validating the ecological concerns associated with this mining region.

The soils in orchards near the pyrite mines of Aljustrel show high contamination levels of arsenic (As), copper (Cu), lead (Pb), and zinc (Zn) [118]. Similarly [119], examined several sites across the Iberian Pyritic Belt, identifying Aljustrel as the location with the highest total concentrations of As, Cu, Pb, and Zn. These concentrations exceed the soil quality guidelines established by the Portuguese Environment Agency, further highlighting the severe contamination in this mining area.

All these studies corroborate our findings, demonstrating significant contamination in the area and confirming that the results obtained from the LUCAS samples do not accurately reflect the environmental reality of the study site. The limitations in data resolution and sampling density in the LUCAS dataset render it inadequate for assessing contamination risks at this scale. Therefore, the use of such low-resolution data is not recommended for studies requiring precise environmental assessments.

## 5 Conclusions

Low-resolution and wide-coverage maps, such as those provided by LUCAS, offer a general overview of environmental issues but lack the necessary detail for precise contamination assessments. In our study, pollution indices were calculated using soil samples, and many of these samples were found to be contaminated. However, when applying the same indices to LUCAS data, these areas of contamination were not detected adequately. This discrepancy highlights a significant limitation of using low-resolution data like LUCAS for accurate contamination assessments.

In contrast, individual indices only effectively identify uncontaminated areas for specific elements and indices. However, this limits the primary goal of identifying and assessing contamination hotspots. The failure to detect contamination in crucial areas suggests that the use of low-resolution maps is inadequate for risk evaluation purposes.

Our analysis shows that increasing the spatial resolution of these maps does not yield significantly better results because the underlying data remain the same as those used to generate the original 1 km resolution maps. Simply increasing pixel size is insufficient to improve accuracy. Instead, the quality and resolution of the maps are primarily determined by the sampling design and the density of sampling points.

Therefore, the traditional approach, based on direct sampling and analysis, remains the most reliable for a comprehensive and accurate contamination study. Sampling-free methods, such as those using interpolated raster data, cannot replace the depth and precision of field-based studies, particularly in areas with high environmental risk.

## Declaration of generative ai and ai-assisted technologies in the writing process

During the preparation of this work, the authors used OpenAI's ChatGPT in order to improve the clarity, language and overall flow of certain sections of the manuscript. After using this tool/service, the author(s) reviewed and edited the content as needed and take(s) full responsibility for the content of the publication.

## CRediT authorship contribution statement

**Silvia Aparisi-Navarro:** Writing – original draft, Validation, Software, Methodology, Investigation, Formal analysis, Data curation, Conceptualization, Resources. **Maria Moncho-Santonja:** Validation, Software, Investigation, Formal analysis. **Beatriz Defez:** Writing – review & editing, Validation, Supervision, Funding acquisition. **Carla Candeias:** Writing – review & editing, Validation, Resources, Data curation, Conceptualization. **Fernando Rocha:** Writing – review & editing, Supervision. **Guillermo Peris-Fajarnés:** Writing – review & editing, Supervision, Funding acquisition.

## Data availability statement

The data supporting this study's findings are openly available at Zenodo at https://doi.org/10.5281/zenodo.11354845.

## Funding SOURCES

This work was supported by “Funding for open access charge: Universitat Politècnica de València". Silvia Aparisi-Navarro was supported by the Research and Development Program (PAID-01-20) of the Universitat Politècnica de València.

## Declaration of competing interest

The authors declare that they have no known competing financial interests or personal relationships that could have appeared to influence the work reported in this paper.
