# Supplementary material for: Exploring environmental risk in soils: Leveraging open data for non-sampling assessment?
Source: Heliyon. 2024 Dec 15;11(1):e41247. doi: 10.1016/j.heliyon.2024.e41247 (PMC11730565; doi:10.1016/j.heliyon.2024.e41247)
Supplement: Multimedia component 1 [file mmc1.docx]

**FIGURES**


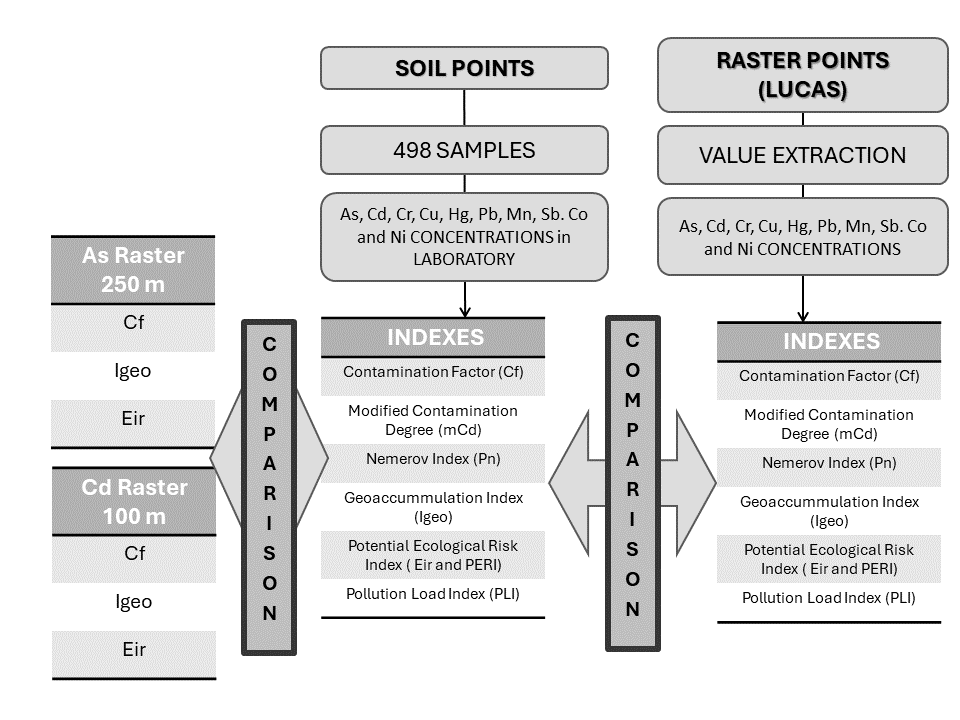


Figure 1. Working Scheme


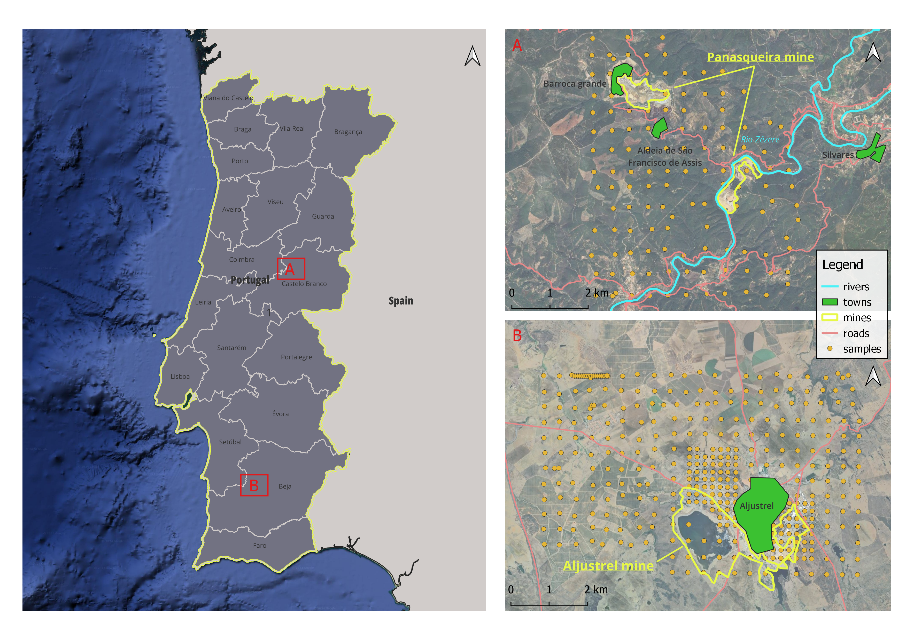


Figure 2. Location of the study areas A) Panasqueira Mine B) Aljustrel Mine

Figure 3. Results of the geoaccumulation index for soil points


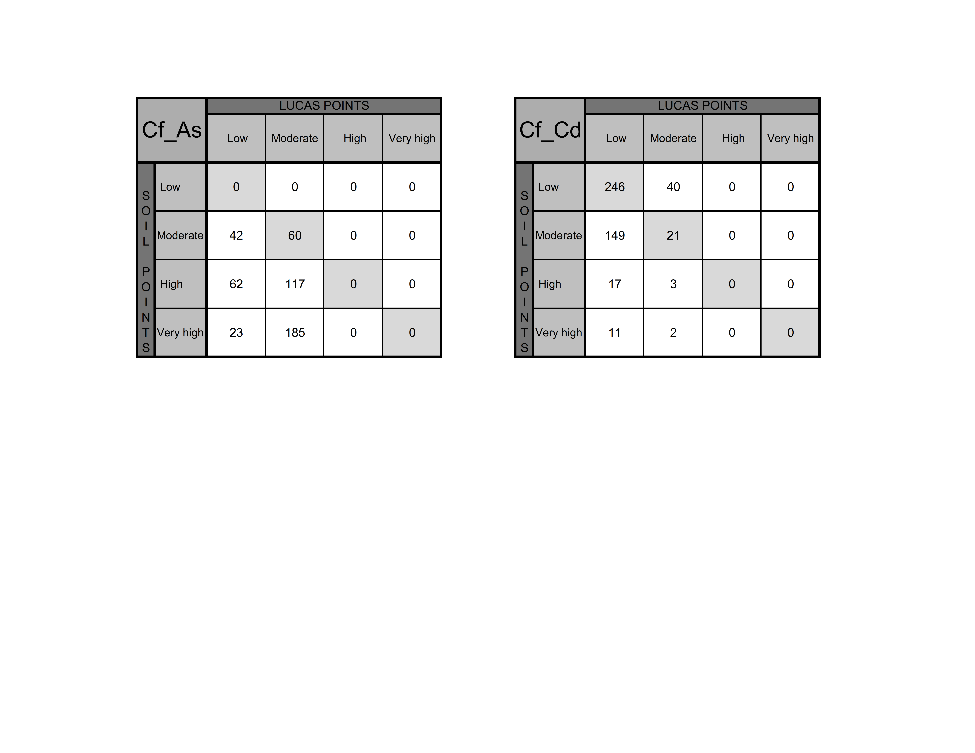

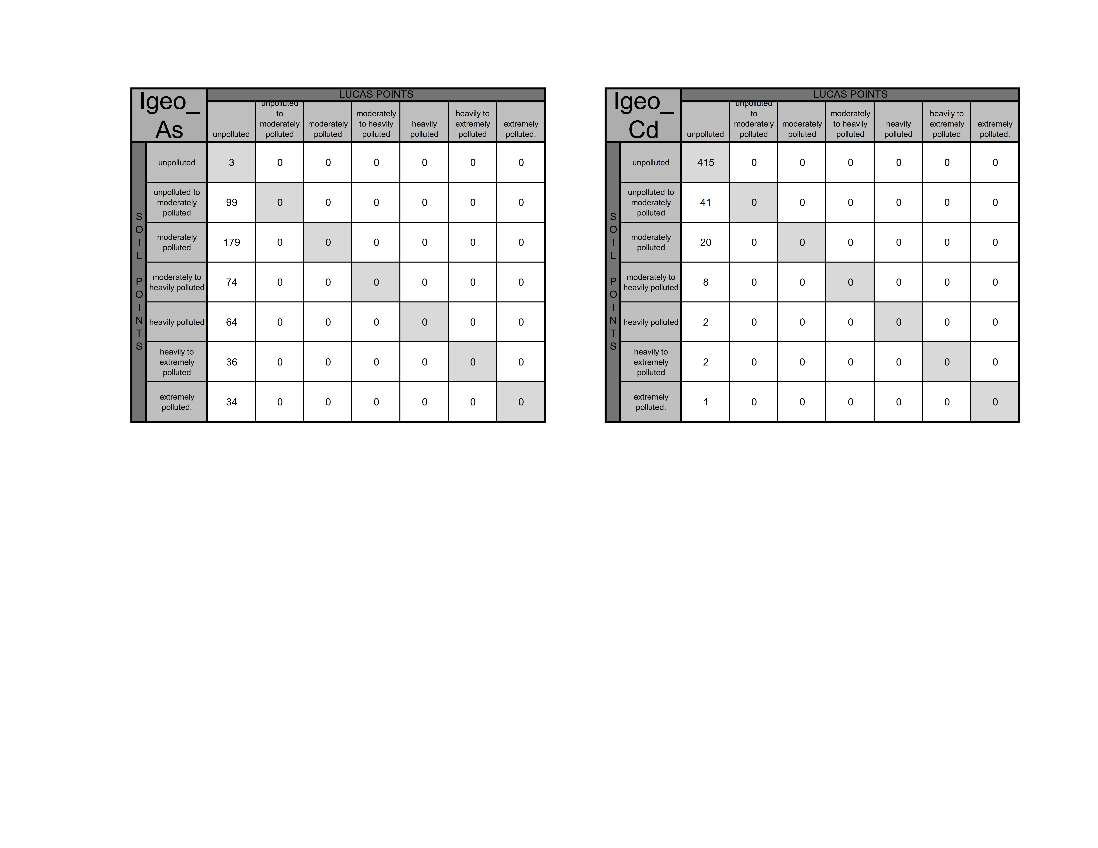


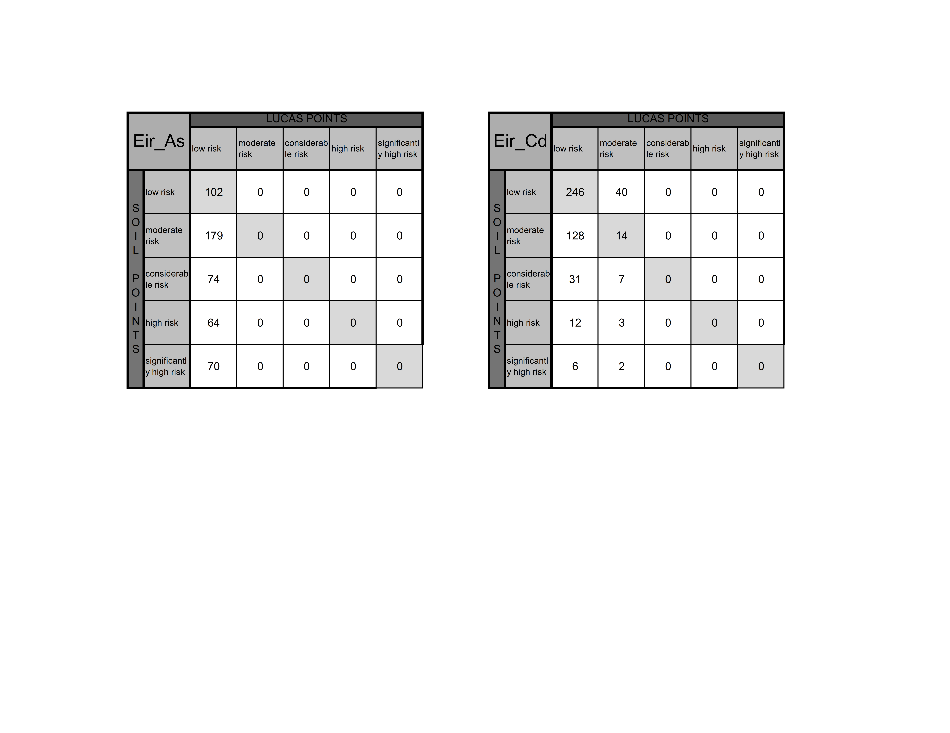


Figure 4. Contamination factor, Geoaccummulation Index and potential risk index of a single element comparison matrices for Arsenic (250 m) and Cadmium (100 m)

**TABLES**

Table 1. Interpretation of pollution indices

| *Index* | *Value* | *Interpetration* |
| --- | --- | --- |
| *Contamination Factor (C_f_)* | *C_f_ < 1* | *Low* |
|  | *1 ≤ C_f_ < 3* | *Moderate* |
|  | *3 ≤ C_f_ < 6* | *High* |
|  | *6 ≤ C_f_* | *Very high* |
| *Modified Contamination Degree (mCd)* | *mCd < 1.5* | *Nil to very low degree of contamination* |
|  | *1.5 ≤ mCd < 2* | *Low degree of contamination* |
|  | *2 ≤ mCd < 4* | *Moderate degree of contamination* |
|  | *4 ≤ mCd < 8* | *High degree of contamination* |
|  | *8 ≤ mCd < 16* | *Very high degree of contamination* |
|  | *16 ≤ mCd < 32* | *Extremely high degree of contamination* |
|  | *mCd ≥ 32* | *Ultra high degree of contamination* |
| *Nemerow Index (Pn)* | *Pn ≤ 0.7* | *Safety domain* |
|  | *0.7 < Pn ≤ 1* | *Precaution domain* |
|  | *1 < Pn ≤ 2* | *Slightly polluted domain* |
|  | *2 < Pn ≤ 3* | *Moderatly polluted domain* |
|  | *Pn > 3* | *Seriously polluted domain* |
| *Geoaccumulation Index (Igeo)* | *Igeo ≤ 0* | *Unpolluted* |
|  | *0 ≤ Igeo < 1* | *Unpolluted to moderately polluted* |
|  | *1 ≤ Igeo < 2* | *Moderately polluted* |
|  | *2 ≤ Igeo < 3* | *Moderately to heavily polluted* |
|  | *3 ≤ Igeo < 4* | *Heavily polluted* |
|  | *4 ≤ Igeo < 5* | *Heavily to extremely polluted* |
|  | *Igeo ≥ 5* | *Extremely polluted.* |
| *Pollution Load Index* | *PLI > 1* | *Polluted* |
|  | *PLI = 1* | *Baseline levels of pollution* |
|  | *PLI < 1* | *Not polluted* |

Table 2. Interpretation of the Potential Ecological Risk Index (PERI)

| *Clasification* | *Value* | *New calculated values for 9 elements* |
| --- | --- | --- |
| *low risk* | *RI < 150* | *RI < 80* |
| *moderate risk* | *150 RI < 300* | *80 RI < 160* |
| *considerable risk* | *300 RI < 600* | *160 RI < 320* |
| *high risk* | *>= RI 600* | *>= RI 320* |

Table 3. % Success rate in classification for individual indices

| % success rate in classification | Cf | Igeo | Eir |
| --- | --- | --- | --- |
| Cu | 19 | 48 | 87 |
| Pb | 7 | 23 | 84 |
| Ni | 74 | 58 | 100 |
| Co | 34 | 59 | 100 |
| Mn | 53 | 61 | 100 |
| As | 0 | 1 | 21 |
| Cd | 58 | 85 | 58 |
| Sb | 18 | 24 | 55 |
| Cr | 89 | 100 | 52 |

Table 4. Success rate in classification for complex indices

| Index | % Success rate in clasification |
| --- | --- |
| Mcd | 15,75% |
| Pn | 2,45% |
| RI | 5% |
| PLI | 18% |

**SUPPLEMENTARY MATERIAL**

Content

[S1. Contamination factor SOIL points 2](#_Toc164170376)

[S2. Contamination factor LUCAS points 2](#_Toc164170377)

[S3. Modified Contamination Degree SOIL points 2](#_Toc164170378)

[S4. Nemerow Index SOIL points 2](#_Toc164170379)

[S5. Nemerow Index LUCAS points 3](#_Toc164170380)

[S6. Geoaccumulation Index SOILPOINTS 3](#_Toc164170381)

[S7. Geoaccumulation Index LUCAS POINTS 3](#_Toc164170382)

[S8. Potential ecological risk index of a single element (Eir) SOIL points 3](#_Toc164170383)

[S9. Potential ecological risk index of a single element (Eir) LUCAS points 3](#_Toc164170384)

[S10. Polution load index SOIL points 4](#_Toc164170385)

[S11. Polution load index LUCAS points 4](#_Toc164170386)

[F1. Factor contamination comparison matrices 4](#_Toc164170387)

[F2. Geoaccumulation index comparison matrices 5](#_Toc164170388)

[F3. Eir comparison matrices 5](#_Toc164170389)

[F4. Modified contamination Degree comparison matrix 6](#_Toc164170390)

[F5. Nemerow Index comparison matrix 6](#_Toc164170391)

[F6. IR comparison matrix 6](#_Toc164170392)

[F7. Pollution load index comparison matrix 7](#_Toc164170393)

[S12. Contamination Factor points As 250 and points Cd 100 7](#_Toc164170394)

[S13.Geoaccumulation index As 250 and Cd 100 7](#_Toc164170395)

[S14. Potential ecological risk index of a single element As 250 and Cd 100 7](#_Toc164170396)

## S1. Contamination factor SOIL points

| Contamination Factor (Cf) | Cu | Pb | Ni | Co | Mn | As | Cd | Sb | Cr |
| --- | --- | --- | --- | --- | --- | --- | --- | --- | --- |
| Cf < 1 Low | 93 | 35 | 144 | 173 | 189 | 0 | 371 | 102 | 433 |
| % | *19,0* | *7,2* | *29,4* | *35,4* | *38,7* | *0,0* | *75,9* | *20,9* | *88,5* |
| 1 ≤ Cf < 3 Moderate | 291 | 299 | 337 | 303 | 261 | 128 | 90 | 71 | 56 |
| % | *59,5* | *61,1* | *68,9* | *62,0* | *53,4* | *26,2* | *18,4* | *14,5* | *11,5* |
| 3 ≤ Cf < 6 High | 39 | 79 | 8 | 12 | 22 | 155 | 15 | 184 | 0 |
| % | *8,0* | *16,2* | *1,6* | *2,5* | *4,5* | *31,7* | *3,1* | *37,6* | *0,0* |
| 6 ≤ Cf Very high | 66 | 76 | 0 | 1 | 17 | 206 | 13 | 132 | 0 |
| % | *13,5* | *15,5* | *0,0* | *0,2* | *3,5* | *42,1* | *2,7* | *27,0* | *0,0* |

## S2. Contamination factor LUCAS points

| Contamination Factor (Cf): | Cu | Pb | Ni | Co | Mn | As | Cd | Sb | Cr |
| --- | --- | --- | --- | --- | --- | --- | --- | --- | --- |
| Cf < 1 Low | 489 | 489 | 74 | 489 | 303 | 403 | 489 | 489 | 489 |
| % | *100,00* | *100,00* | *15,1* | *100,00* | *61,96* | *82,41* | *100,00* | *100,00* | *100,00* |
| 1 ≤ Cf < 3 Moderate | 0 | 0 | 415 | 0 | 186 | 86 | 0 | 0 | 0 |
| % | *0,00* | *0,00* | *84,87* | *0,00* | *38,04* | *17,59* | *0,00* | *0,00* | *0,00* |
| 3 ≤ Cf < 6 High | 0 | 0 | 0 | 0 | 0 | 0 | 0 | 0 | 0 |
| % | *0,00* | *0,00* | *0,00* | *0,00* | *0,00* | *0,00* | *0,00* | *0,00* | *0,00* |
| 6 ≤ Cf Very high | 0 | 0 | 0 | 0 | 0 | 0 | 0 | 0 | 0 |
| % | *0,00* | *0,00* | *0,00* | *0,00* | *0,00* | *0,00* | *0,00* | *0,00* | *0,00* |

## S3. Modified Contamination Degree SOIL points

| modificated contamination degree = mCD | n | % |
| --- | --- | --- |
| mCd < 1,5—Nil to very low degree of contamination | 77 | 15,75 |
| 1,5 < mCd < 2—Low degree of contamination | 123 | 25,15 |
| 2 < mCd < 4—Moderate degree of contamination | 177 | 36,20 |
| 4 < mCd < 8—High degree of contamination | 57 | 11,66 |
| 8 < mCd < 16—Very high degree of contamination | 20 | 4,09 |
| 16 < mCd < 32—Extremely high degree of contamination | 11 | 2,25 |
| mCd > 32—Ultra high degree of contamination | 24 | 4,91 |
| TOTAL | 489 | 100 |

## S4. Nemerow Index SOIL points

| **Value** | **classification** | | | **n** | **%** |
| --- | --- | --- | --- | --- | --- |
| Pn≤0.7 | safety domain | | | 0 | 0,0 |
| 0.7<Pn≤1 | precaution domain | | | 0 | 0,0 |
| 1<Pn≤2 | slightly polluted domain | | | 12 | 2,5 |
| 2<Pn≤3 | moderatly polluted domain | | | 91 | 18,6 |
| Pn > 3 | seriously polluted domain | | | 386 | 78,9 |
|  |  |  |  | 489 | 100 |

## S5. Nemerow Index LUCAS points

| **Value** | **classification** | | | **n** | **%** |
| --- | --- | --- | --- | --- | --- |
| Pn≤0.7 | safety domain | | | 0 | 0,0 |
| 0.7<Pn≤1 | precaution domain | | | 111 | 22,7 |
| 1<Pn≤2 | slightly polluted domain | | | 378 | 77,3 |
| 2<Pn≤3 | moderatly polluted domain | | | 0 | 0,0 |
| Pn > 3 | seriously polluted domain | | | 0 | 0,0 |
|  |  |  |  | 489 | 100 |

## S6. Geoaccumulation Index SOILPOINTS

| Igeo Value | **Cu** | **Pb** | **Ni** | **Co** | **Mn** | **As** | **Cd** | **Sb** | **Cr** |
| --- | --- | --- | --- | --- | --- | --- | --- | --- | --- |
| Igeo ≤ 0 | 239 | 112 | 294 | 294 | 300 | 3 | 415 | 115 | 488 |
| 0 < Igeo ≤ 1 | 145 | 222 | 187 | 182 | 150 | 125 | 46 | 58 | 1 |
| 1 < Igeo ≤ 2 | 39 | 79 | 8 | 12 | 22 | 155 | 15 | 184 | 0 |
| 2 < Igeo ≤ 3 | 26 | 32 | 0 | 0 | 10 | 74 | 8 | 55 | 0 |
| 3 < Igeo ≤ 4 | 24 | 10 | 0 | 1 | 5 | 63 | 2 | 30 | 0 |
| 4 < Igeo ≤ 5 | 10 | 12 | 0 | 0 | 1 | 35 | 2 | 13 | 0 |
| Igeo > 5 | 6 | 22 | 0 | 0 | 1 | 34 | 1 | 34 | 0 |

## S7. Geoaccumulation Index LUCAS POINTS

| Value | **Cu** | **Pb** | **Ni** | **Co** | **Mn** | **As** | **Cd** | **Sb** | **Cr** |
| --- | --- | --- | --- | --- | --- | --- | --- | --- | --- |
| Igeo _ 0 | 489 | 489 | 167 | 489 | 489 | 489 | 489 | 489 | 489 |
| 0 < Igeo < 1 | 0 | 0 | 322 | 0 | 0 | 0 | 0 | 0 | 0 |
| 1 < Igeo < 2 | 0 | 0 | 0 | 0 | 0 | 0 | 0 | 0 | 0 |
| 2 < Igeo < 3 | 0 | 0 | 0 | 0 | 0 | 0 | 0 | 0 | 0 |
| 3 < Igeo < 4 | 0 | 0 | 0 | 0 | 0 | 0 | 0 | 0 | 0 |
| 4 < Igeo < 5 | 0 | 0 | 0 | 0 | 0 | 0 | 0 | 0 | 0 |
| Igeo > 5 | 0 | 0 | 0 | 0 | 0 | 0 | 0 | 0 | 0 |

## S8. Potential ecological risk index of a single element (Eir) SOIL points

| **Value** | **clasification** | **Cu** | **Pb** | **Ni** | **Co** | **Mn** | **As** | **Cd** | **Sb** | **Cr** |
| --- | --- | --- | --- | --- | --- | --- | --- | --- | --- | --- |
| Eir< 30 | Low risk | 423 | 413 | 489 | 488 | 488 | 128 | 371 | 270 | 489 |
| 30< Eir< 60 | Moderate Risk | 26 | 32 | 0 | 0 | 1 | 155 | 67 | 116 | 0 |
| 60< Eir< 120 | Considerable risk | 24 | 10 | 0 | 1 | 0 | 74 | 31 | 44 | 0 |
| 120 <Eir< 240 | high risk | 10 | 12 | 0 | 0 | 0 | 63 | 12 | 16 | 0 |
| Eir> 240 | significantly high risk | 6 | 22 | 0 | 0 | 0 | 69 | 8 | 43 | 0 |

## S9. Potential ecological risk index of a single element (Eir) LUCAS points

| **Value** | **clasification** | **Cu** | **Pb** | **Ni** | **Co** | **Mn** | **As** | **Cd** | **Sb** | **Cr** |
| --- | --- | --- | --- | --- | --- | --- | --- | --- | --- | --- |
| Eir< 30 | Low risk | 489 | 489 | 489 | 489 | 489 | 489 | 489 | 489 | 254 |
| 30< Eir< 60 | Moderate Risk | 0 | 0 | 0 | 0 | 0 | 0 | 0 | 0 | 235 |
| 60< Eir< 120 | Considerable risk | 0 | 0 | 0 | 0 | 0 | 0 | 0 | 0 | 0 |
| 120 <Eir< 240 | high risk | 0 | 0 | 0 | 0 | 0 | 0 | 0 | 0 | 0 |
| Eir> 240 | significantly high risk | 0 | 0 | 0 | 0 | 0 | 0 | 0 | 0 | 0 |

## S10. Polution load index SOIL points

| Value | PLI | % |
| --- | --- | --- |
| Polluted (PLI > 1) | 399 | 81,59 |
| Baseline levels of pollution (PLI = 1 | 0 | 0 |
| Not polluted (PLI < 1). | 90 | 18,41 |
| total | 489 | 100 |

## S11. Polution load index LUCAS points

| Value | PLI | % |
| --- | --- | --- |
| Polluted (PLI > 1) | 0 | 0 |
| Baseline levels of pollution (PLI = 1 | 0 | 0 |
| Not polluted (PLI < 1). | 489 | 100 |
| total | 489 | 100 |

## F1. Factor contamination comparison matrices


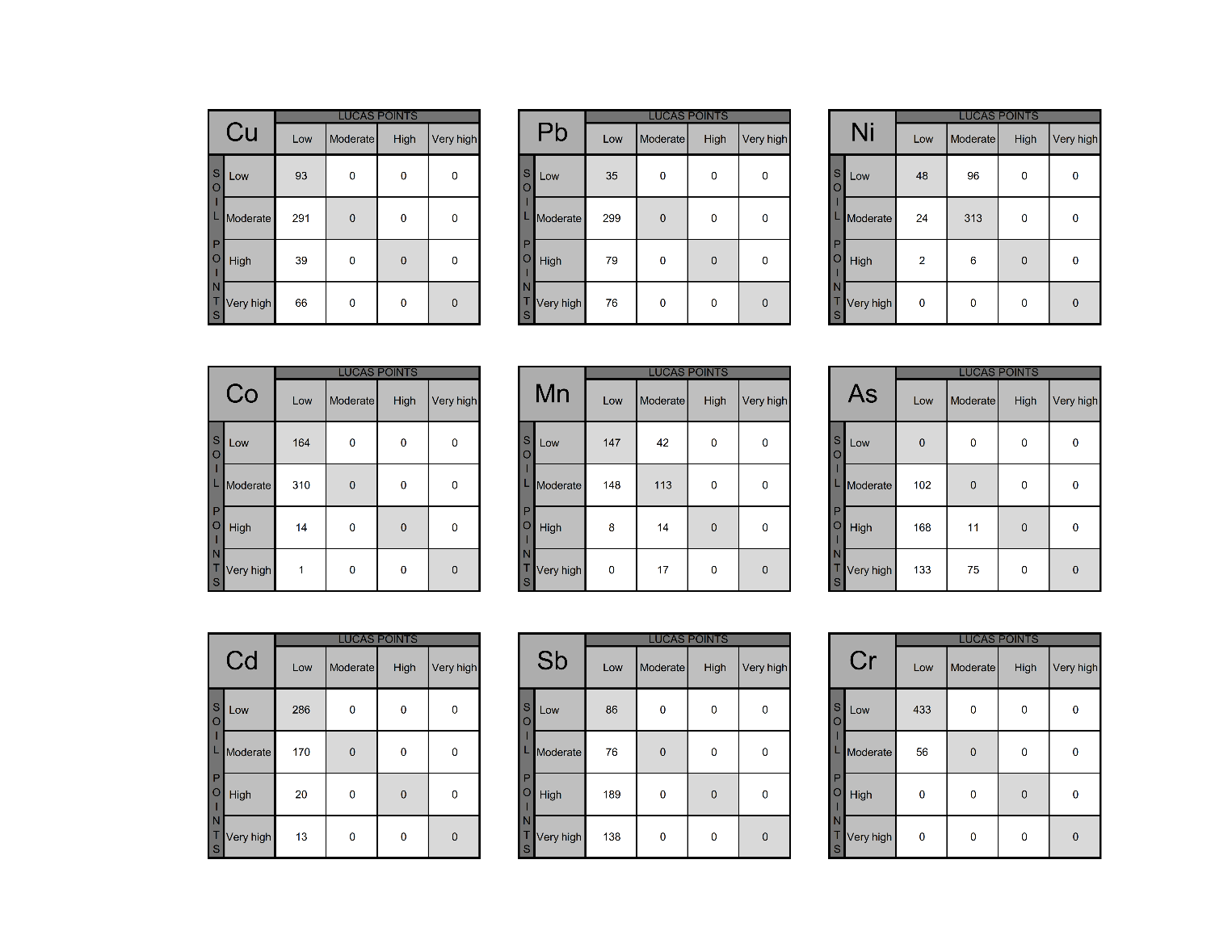


## F2. Geoaccumulation index comparison matrices


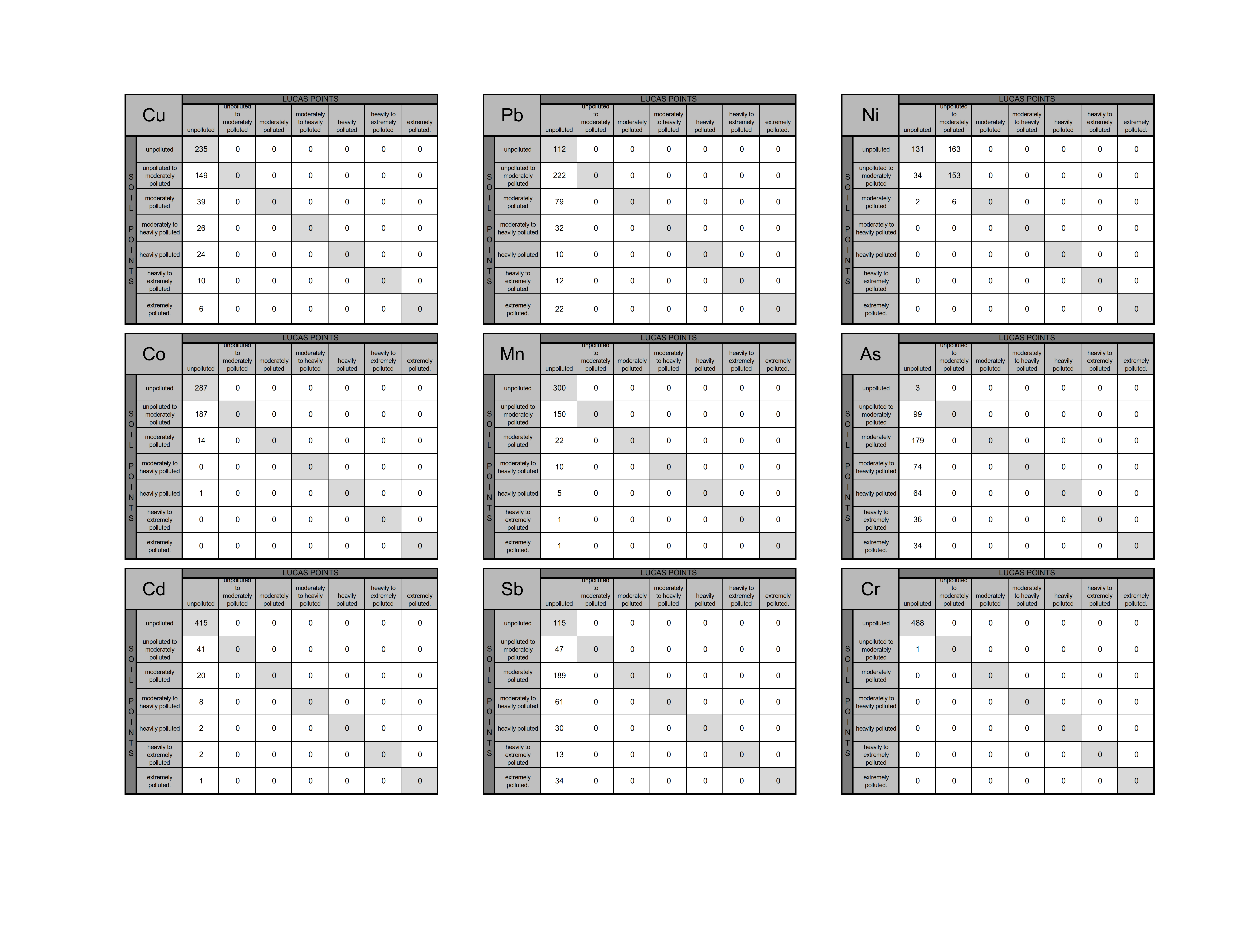


## F3. Eir comparison matrices


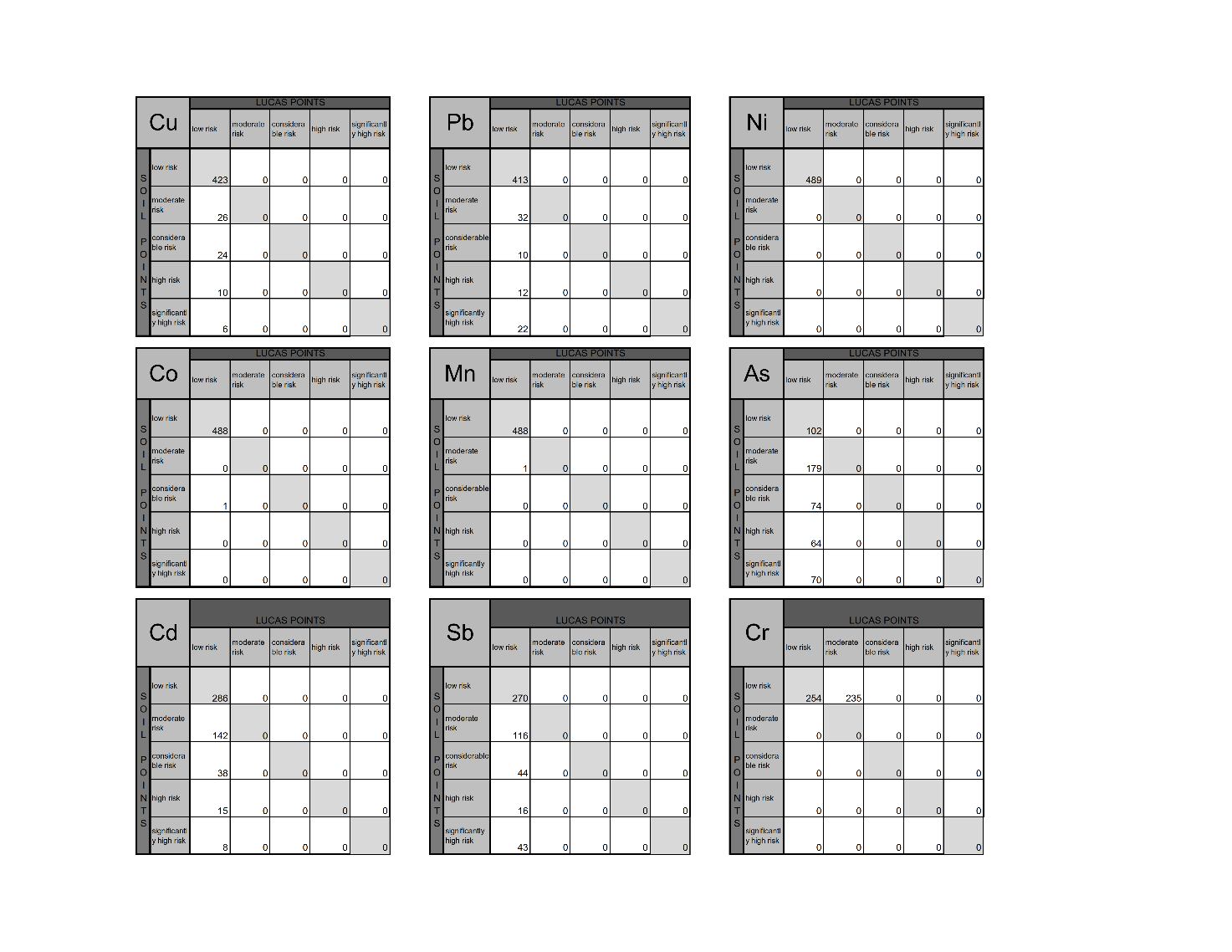


## F4. Modified contamination Degree comparison matrix

## F5. Nemerow Index comparison matrix

## F6. IR comparison matrix

## F7. Pollution load index comparison matrix

## S12. Contamination Factor points As 250 and points Cd 100

| Contamination Factor (EF): | **As** | **Cd** | **As %** | **Cd%** |
| --- | --- | --- | --- | --- |
| EF < 1 Low | 127 | 423 | 26% | 87% |
| 1 ≤ EF < 3 Moderate | 362 | 66 | 74% | 13% |
| 3 ≤ EF < 6 High | 0 | 0 | 0% | 0% |
| 6 ≤ EF Very high | 0 | 0 | 0% | 0% |

## S13.Geoaccumulation index As 250 and Cd 100

| Igeo | **Igeo As** | **Igeo Cd** |
| --- | --- | --- |
| Igeo _ 0 | 489 | 489 |
| 0 < Igeo < 1 | 0 | 0 |
| 1 < Igeo < 2 | 0 | 0 |
| 2 < Igeo < 3 | 0 | 0 |
| 3 < Igeo < 4 | 0 | 0 |
| 4 < Igeo < 5 | 0 | 0 |
| Igeo > 5 | 0 | 0 |

## S14. Potential ecological risk index of a single element As 250 and Cd 100

| Value EIR | **Eir As** | **Eir Cd** |
| --- | --- | --- |
| Eir< 30 | 489 | 423 |
| 30< Eir< 60 | 0 | 66 |
| 60< Eir< 120 | 0 | 0 |
| 120 <Eir< 240 | 0 | 0 |
| Eir> 240 | 0 | 0 |
